# Prediction of pCR based on clinical-radiomic model in patients with locally advanced ESCC treated with neoadjuvant immunotherapy plus chemoradiotherapy

**DOI:** 10.3389/fonc.2024.1350914

**Published:** 2024-03-20

**Authors:** Xiaohan Wang, Guanzhong Gong, Qifeng Sun, Xue Meng

**Affiliations:** ^1^ Department of Radiation Oncology, Shandong Cancer Hospital and Institute, Shandong First Medical University and Shandong Academy of Medical Science, Jinan, China; ^2^ Department of Radiotherapy, Shandong Cancer Hospital and Institute, Shandong First Medical University and Shandong Academy of Medical Science, Jinan, China; ^3^ Department of Thoracic Surgery, Shandong Provincial Hospital Affiliated to Shandong First Medical University, Jinan, China

**Keywords:** esophageal squamous cell carcinoma, immunotherapy, neoadjuvant therapy, radiomics, inflammatory biomarkers

## Abstract

**Background:**

The primary objective of this research is to devise a model to predict the pathologic complete response in esophageal squamous cell carcinoma (ESCC) patients undergoing neoadjuvant immunotherapy combined with chemoradiotherapy (nICRT).

**Methods:**

We retrospectively analyzed data from 60 ESCC patients who received nICRT between 2019 and 2023. These patients were divided into two cohorts: pCR-group (N = 28) and non-pCR group (N = 32). Radiomic features, discerned from the primary tumor region across plain, arterial, and venous phases of CT, and pertinent laboratory data were documented at two intervals: pre-treatment and preoperation. Concurrently, related clinical data was amassed. Feature selection was facilitated using the Extreme Gradient Boosting (XGBoost) algorithm, with model validation conducted via fivefold cross-validation. The model’s discriminating capability was evaluated using the area under the receiver operating characteristic curve (AUC). Additionally, the clinical applicability of the clinical-radiomic model was appraised through decision curve analysis (DCA).

**Results:**

The clinical-radiomic model incorporated seven significant markers: postHALP, ΔHB, post-ALB, firstorder_Skewness, GLCM_DifferenceAverage, GLCM_JointEntropy, GLDM_DependenceEntropy, and NGTDM_Complexity, to predict pCR. The XGBoost algorithm rendered an accuracy of 0.87 and an AUC of 0.84. Notably, the joint omics approach superseded the performance of solely radiomic or clinical model. The DCA further cemented the robust clinical utility of our clinical-radiomic model.

**Conclusion:**

This study successfully formulated and validated a union omics methodology for anticipating the therapeutic outcomes of nICRT followed by radical surgical resection. Such insights are invaluable for clinicians in identifying potential nICRT responders among ESCC patients and tailoring optimal individualized treatment plans.

## 1 Introduction

Esophageal cancer (EC), a malignant tumor of the digestive tract, is characterized predominantly by progressive dysphagia. Globally, it holds the seventh spot in incidence, with 604,000 new cases reported, and is sixth in mortality, accounting for 544,000 deaths annually (1). As per recent data from the China National Cancer Center, esophageal cancer’s prevalence places it sixth, while its mortality rate occupies the fourth position. Notably, the esophageal squamous cell carcinoma (ESCC) subtype is predominant in China, underscoring its significance in the broader context of EC (2, 3).

The prevailing therapeutic paradigm for patients with locally advanced but resectable esophageal cancer is neoadjuvant chemoradiotherapy (nCRT), subsequently followed by definitive surgical intervention (4, 5). With the development of immunotherapy, the concurrent use of immune checkpoint inhibitors (ICIs) with chemotherapy has shown marked improvement in survival outcomes for those with advanced or metastatic disease states (6–10). Preliminary trials investigating the combination of ICIs with neoadjuvant chemoradiotherapy for resectable EC have been encouraging (11, 12). The pCR rate of the nICRT group was 0.48, which was slightly higher than that of the nCRT group (pCR rate: 0.28–0.49) (4, 13–16). However, the inter-individual variability in response to nICRT is significant, highlighting the unmet need to identify robust predictive biomarkers for pCR.

Several clinical factors, such as stage, gender, gross target volume (GTV), etc., have proven to be associated with treatment outcomes (17–20). In addition, quantitative imaging biomarkers have been of interest for the application in clinical prediction models (21–23). Radiomics is a non-invasive technique that involves the extraction of quantitative radiomics features (RFs) from conventional medical images, the selection of features by using particular methods, and the analysis of the correlation between the clinical data and clinical outcomes, which may finally support clinical decision-making (24, 25). Notably, the radiomic model based on CT to predict pCR after neoadjuvant therapy has a good power of prediction, especially in ESCC patients, with a high-performing level and good discrimination ability. In a study by Yang et al. (26), three CT-based radiomic models were used to predict pCR in ESCC patients after nCRT in both the training (AUC, 0.84-0.86) and test cohorts (AUC, 0.71-0.79). In addition, peritumoral features which serve as powerful prognostic indicators can be used to construct radiomic models. Hu et al. (27) found that the combination of intratumoral and peritumoral features to establish a joint radiomic model based on CT demonstrated good performance on identification and better prediction of pCR. There are some previous studies that built predictive models based on clinical risk factors and radiomics features. Gong et al. (28) found that a model combining radiomic features of contrast-enhanced CT and clinical characteristics could predict the recurrence rate of EC among patients treated with definitive chemoradiotherapy.

Despite these advancements, a limitation in many radiomics studies is the inclusion of both adenocarcinoma and squamous cell carcinoma patients. Given that the pCR rates might be distinctively higher in ESCC patients (14, 29) and considering that ESCC represents about 90% of EC cases (30), our study focuses on this subset. We leverage a combination of radiomic features, clinical data, and hematological markers to predict pCR in ESCC patients post-nICRT. This comprehensive approach aims to furnish clinicians with insights at the onset of treatment, enabling a more personalized therapeutic strategy.

## 2 Materials and methods

### 2.1 Patient selection and demographics

Patients diagnosed with ESCC, who underwent radical surgical resection following nICRT at Shandong Cancer Hospital and Shandong Provincial Hospital between January 2019 and May 2023, were retrospectively enrolled. Inclusion criteria encompassed a confirmed ESCC diagnosis with an available gold standard postoperative pathology report, receipt of nICRT, undergoing a contrast-enhanced CT scan within one month preceding both the neoadjuvant therapy and surgery, and having comprehensive clinical records. Exclusion criteria included the presence of other synchronous malignancies, suboptimal CT quality impeding diagnosis, or an incomplete diagnostic and therapeutic trajectory within institutions. Staging was in line with the eighth edition of the American Joint Committee on Cancer (AJCC)’s tumor-node-metastasis (TNM) staging for EC (31). This study adhered to the Declaration of Helsinki and received approval from the institutional review board of Shandong Cancer Hospital and Institute.

### 2.2 Treatment

All patients were treated with paclitaxel combined with platinum. Due to the ongoing exploration of neoadjuvant chemoradiotherapy combined with immunotherapy for esophageal cancer, the use of immunotherapy drugs follows clinical trials. The doses adjustments of regimens were decided by the doctor based on the guidelines of the National Comprehensive Cancer Network or the Chinese Society of Clinical Oncology. Regarding radiation techniques, intensity-modulated radiotherapy (IMRT) was used for thoracic radiotherapy. At present, there is no specific regulation on the target volume for neoadjuvant radiotherapy for esophageal cancer internationally. It is recommended to follow the principle of involving field irradiation in radical radiotherapy. The organs at risk (OARs) and target volumes were defined based on the Radiation Therapy and Oncology Group guidelines for esophageal cancer. The primary tumor and the positive lymph nodes were included in the GTV, named GTVp and GTVn, respectively. The clinical target volume (CTV) was expanded from GTVp with a 0.5-0.6 cm radially, and a 3 cm cranio-caudally, and included all the positive regional nodal regions at diagnosis. The planning target volume (PTV) was increased by 0.5–0.8 cm from the CTV (32, 33). The radiation therapy plans were made to ensure adequate coverage of the prescribed radiation dose was at least ninety-five percent of the PTV. The total radiation dose ranges from 40 Gy to 50.4 Gy, at 1.8 Gy to 2 Gy per fraction once daily (five fractions per week) (34, 35).

### 2.3 Information collection and follow-up

Clinicopathological characteristics including age, sex, smoking and drinking status, diabetes and hypertension history, body mass index (BMI), TNM stage, pathological differentiation, tumor location, length of the diseased esophagus, cycles of chemotherapy combined with immunotherapy, and radiotherapy dose were extracted from the patients’ medical records. The laboratory data included lactate dehydrogenase (LDH), albumin (ALB), prealbumin (PALB), absolute white blood cell count (WBC), absolute neutrophil count (ANC), absolute lymphocyte count (ALC), absolute monocyte count (AMC), absolute platelet count (APC), hemoglobin count (HB) and NLR, MLR, PLR, HALP, SII, SIRI, and PNI. The NLR, MLR, PLR, HALP, SII, SIRI, and PNI were calculated using the following formulas: NLR = ANC/ALC, MLR = AMC/ALC, PLR = APC/ALC, HALP=HB×ALB×ALC/APC, SII = APC×ANC/ALC, SIRI=AMC×ANC/ALC, PNI = ALB (g/L) + 5 ×ALC (109/L). These immune-related inflammatory biomarkers (IBs) were calculated during two periods: roughly a week pre-treatment (pre-IBs) and a week preoperation (post-IBs). The difference between these markers over these intervals (delta-IBs) was also calculated. Patients were categorized into either the pCR group or the non-pCR group based on the absence (ypT0N0) or presence of viable tumor cells in the primary tumor area and all excised lymph nodes, respectively (36).

### 2.4 Imaging acquisition and radiomic segmentation

A 64-layer spiral CT scanner (Definition AS+, Siemens SOMATOM) was deployed for CT scans. Parameters included a 5.0 mm slice thickness, 120 kV tube voltage, and 220 mA tube current. An iodinated contrast agent (300 mg/mL) was administered at 1.5 ml/kg body mass at a 2 mL/s rate. Digital contrast-enhanced CT scans in digital imaging and communications in medicine (DICOM) format were retrieved from the picture archiving and communication systems (PACS) and subsequently processed using the open-source radiomics extraction toolkit.

For quantitative imaging analysis, the primary gross tumor was defined as lesions with esophageal wall thickening > 5 mm or lumen occlusion diameter > 10 mm and excluding intraluminal gas and oral contrast agents and selected as the region of interest (ROI) (37), with normal structures and metastatic lymph nodes omitted. ROI parameters were set at a window width of 400 and a window level of 40. Initially, for the CT images of the arterial phase prior to treatment, the delineation of ROI-B was assisted using medicalmind software for edge detection, followed by manual tracing and correction along the primary esophageal tumor contour. Subsequently, CT images from different phases, both before and after treatment, were aligned with the arterial phase CT images before treatment using projection transformation. The contour of the ROI from the pre-treatment arterial phase CT images was then projected onto these CT images. Following this, the original radiation oncologist manually adjusted the contours to account for changes due to tumor shrinkage after treatment, ensuring consistency in the anatomical range from cranial to caudal for depicting ROI-A/C/D/E/F. ROI-D/E/F, identified in the preoperative plain, arterial, and venous phases, were designated as areas of the esophagus with visible tumor presence. In cases where no residual tumor was observed after neoadjuvant radiochemotherapy combined with immunotherapy, ROIs were marked at the primary tumor bed location. Experienced radiologists meticulously executed and reviewed all segmentations to ensure precision.

### 2.5 Feature extraction

We extracted the radiomics features from the basal CT before any therapy and the contrast-enhanced CT which is for evaluating efficacy before surgery. Total of 107 RFs based on patient CT images were extracted from each phase using the medmind software, encompassing 14 shape features, 18 first order statistical features, 24 gray level co-occurrence matrix (GLCM), 14 gray level dependence matrix (GLDM), 16 gray level run-length matrix (GLRLM), 16 gray level size zone matrix (GLSZM), and 5 neighbor gray tone difference matrix (NGTDM), detailed at https://pyradiomics.readthedocs.io/en/latest.

### 2.6 Feature selection and model development

The eXtreme Gradient Boosting (XGBoost) algorithm was employed, leveraging its gradient boosting capabilities (38). The importance of each feature was ranked by calculation. In order to choose the relevant features for building the classification model, we recursively removed the features with lower importance to obtain smaller feature subsets, estimated the discriminative abilities of features of the subsets, and selected those features with the greatest discriminative power to enhance the prediction performance. To find the optimal number of features, 5-fold cross-validation was employed to score different feature subsets and select the best scoring set of features. With the selected radiomics features, radiomics models with good prediction performance for pCR were established. By comparing a suite of statistical metrics, including accuracy, precision, and recall, the best radiomics model was selected.

Clinical features were also selected by the XGBoost algorithm to provide the relevant features based on feature importance. Then features with low importance were removed through the recursive elimination method. Clinical features were scored using 5-fold cross-validation on different feature subsets for the selection of best feature subset.

Finally, by combining the RFs and clinical features selected, the XGboost algorithm based on feature importance was used again for screening. The following steps were as the same as above. The prediction abilities of the clinical model, radiomics model, and clinical-radiomic model for pCR were evaluated by the receiver operator characteristic (ROC) curve. The clinical utility of models was ascertained via Decision Curve Analysis (DCA).

### 2.7 Statistical analysis

All statistical evaluations were executed using SPSS Statistics V25.0 (IBM Corporation, Armonk, NY, USA). In our analysis, we distinguished between categorical and continuous variables, employing the Chi-square test and Fisher’s exact test for the categorical variables and choosing between the independent sample t-test (for normally distributed data) or the Mann-Whitney U-test (for data not following a normal distribution) for continuous variables. For the purpose of statistical significance, a p-value below 0.05 was considered indicative of a significant result. Machine-learning analyses were facilitated using the R software (version 3.4.4). The “pROC” packages were employed to draw ROC curves and to evaluate the model performance by the AUC.

## 3 Results

### 3.1 Patient demographics and baseline characteristic

**Figure 1:**
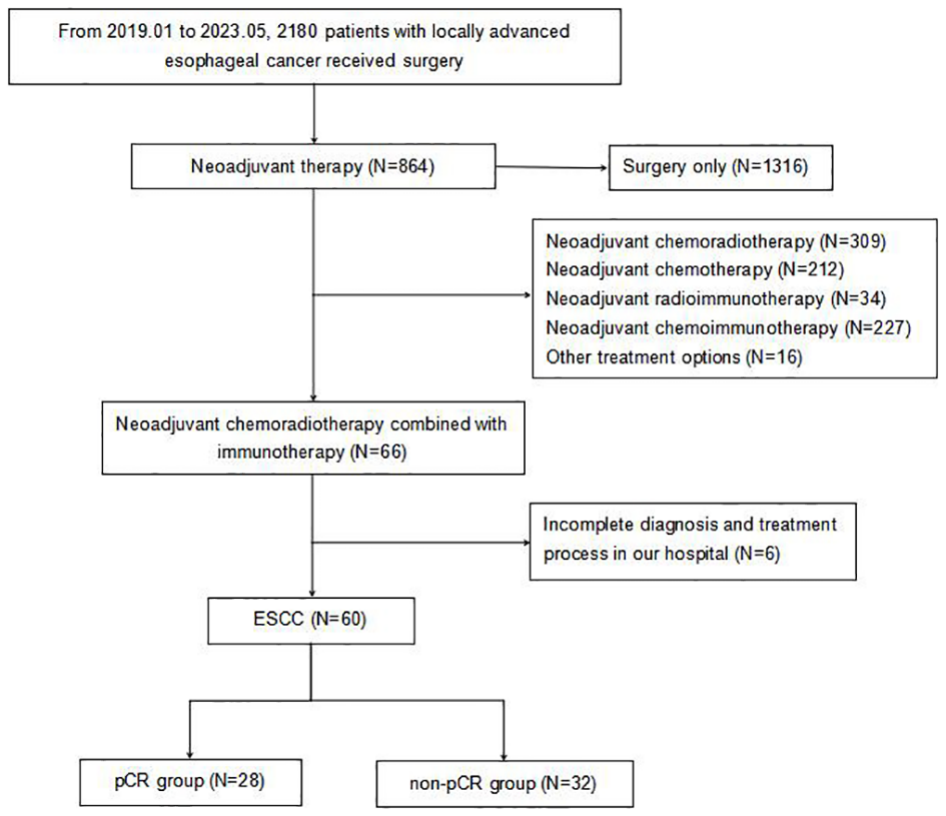
Flowchart of patient enrollment. pCR, pathologic complete response; ESCC, esophageal squamous cell carcinoma.

Initially, 66 patients were considered for the study. However, due to incomplete data on treatment protocols or associated diagnostic materials for 6 individuals, our model was confined to 60 patients, as depicted in **Figure 1**. The baseline attributes for both the pCR and non-pCR groups are outlined in **Table 1**. Of all the patients we enrolled, 66.7% were at stage III, and 33.3% were at stage II. The cohort consisted of 50 males (83.3%) and 10 females (16.7%) with a median age of 59.5 years, ranging from 45 to 73 years. The median tumor length was determined to be 5.5 cm, spanning from 2 to 11 cm. 95% of tumors were located in the middle and lower esophagus. Upon diagnosis, almost all patients with locally advanced ESCC received neoadjuvant therapy within 3 months. A total of 56 (93.4%) patients received at least two cycles of nICRT. Among them, all patients were given paclitaxel combined with platinum, and 50% of patients received SHR1701 immune preparation. 36.7% received tirelizumab and the remaining patients received carrelizumab. 83.3% of patients received radiotherapy exceeding 40Gy, with the majority receiving 41.4Gy/23F. The pCR rate to the neoadjuvant therapy was 46.67%. Both groups showcased no significant disparity in these baseline metrics (P value > 0.05).

**Table 1:** Baseline characteristics of all patients.

Clinical parameter	Total	pCR-group	non-pCR-group	P-value
	N=60	N=28	N=32	
Age				0.365
≤65Y	48 (80.0%)	21 (75.0%)	27 (84.4%)	
>65Y	12 (20.0%)	7 (25.0%)	5 (15.6%)	
Gender				0.563
Male	50 (83.3%)	22 (79.6%)	28 (87.5%)	
Female	10 (16.7%)	6 (21.4%)	4 (12.5%)	
Smoking status				0.139
Former/current	36 (60.0%)	14 (50.0%)	22 (68.7%)	
Never	24 (40.0%)	14 (50.0%)	10 (31.3%)	
Alcohol consumption				0.073
Former/current	31 (51.7%)	11 (39.3%)	20 (62.5%)	
Never	29 (48.3%)	17 (60.7%)	12 (37.5%)	
Diabetes history				0.876
Yes	5 (8.3%)	3 (10.7%)	2 (6.2%)	
No	55 (91.7%)	25 (89.3%)	30 (93.8%)	
Hypertension history				0.744
Yes	14 (23.3%)	6 (21.4%)	8 (25.0%)	
No	46 (76.7%)	22 (78.6%)	24 (75.0%)	
BMI				0.175
<18.5	2 (3.3%)	0 (0.0%)	2 (6.2%)	
18.5~25	32 (53.3%)	13 (46.4%)	19 (59.4%)	
≥25	26 (33.4%)	15 (53.6%)	11 (34.4%)	
Stage T				0.546
T2	6 (10.0%)	4 (14.3%)	2 (6.3%)	
T3	54 (90.0%)	24 (85.7%)	30 (93.7%)	
Stage N				0.668
N0	16 (26.7%)	9 (32.2%)	7 (21.9%)	
N1	30 (50.0%)	13 (46.4%)	17 (53.1%)	
N2	14 (23.3%)	6 (21.4%)	8 (25.0%)	
Clinical stage				0.143
II	20 (33.3%)	12 (42.9%)	8 (25.0%)	
III	40 (66.7%)	16 (57.1%)	24 (75.0%)	
Tumor differentiation				0.066
Gx	28 (46.7%)	17 (60.7%)	11 (34.4%)	
G1 (Well)	3 (5.0%)	2 (7.1%)	1 (3.1%)	
G2 (Moderate)	20 (33.3%)	5 (17.9%)	15 (46.9%)	
G3 (Poor)	9 (15.0%)	4 (14.3%)	5 (15.6%)	
Tumor location				0.130
Upper thoracic	3 (5.0%)	0 (0%)	3 (9.3%)	
Middle thoracic	26 (43.3%)	12 (42.9%)	14 (43.8%)	
Lower thoracic	31 (51.7%)	16 (57.1%)	15 (46.9%)	
Chemotherapy regimen				0.068
Paclitaxel and cisplatin	21 (35.0%)	13 (46.4%)	8 (25.0%)	
Paclitaxel and carboplatin	35 (58.3%)	15 (42.9%)	23 (71.9%)	
Paclitaxel and nedaplatin	4 (6.7%)	23 (10.7%)	1 (3.1%)	
Prescribed dose				0.203
≤40Gy	10 (16.7%)	7 (25.0%)	3 (9.4%)	
>40Gy	50 (83.3%)	21 (75.0%)	29 (90.6%)	
Immunotherapy				0.152
Tirelizumab	22 (36.7%)	14 (50.0%)	8 (25.0%)	
SHR1701	30 (50.0%)	11 (39.3%)	19 (59.4%)	
Karelizumab	8 (13.3%)	3 (10.7%)	5 (15.6%)	
Treatment cycle				0.930
1	4 (6.6%)	3 (10.7%)	2 (6.3%)	
2	51 (85.0%)	23 (82.1%)	27 (84.4%)	
3	4 (6.7%)	2 (7.2%)	2 (6.3%)	
4	1 (1.7%)	0 (0.0%)	1 (3.0%)	

P value was calculated across treatment groups for categorical data using Chi-square test and Fisher’s exact test. pCR, pathologic complete response; BMI, Body Mass Index; T, tumor; N, node.

The comparison of baseline characteristics and treatment specifics between the groups of patients achieving pathological complete response (pCR) and those not achieving pCR revealed no significant differences in most evaluated criteria. This indicates that these initial factors may not significantly influence the rates of pCR observed, supporting the validity of predictive models based on radiomic and clinical data.

Additionally, our thorough analysis explored the relationship between pCR and a wide range of 41 hematological markers in the entire study population (refer to **Supplementary Table 1**). Among these numerous markers, three showed a statistically significant association with pCR rates in the collective group, each presenting p-values below 0.05.

### 3.2 Feature selection

**Figure 2:**
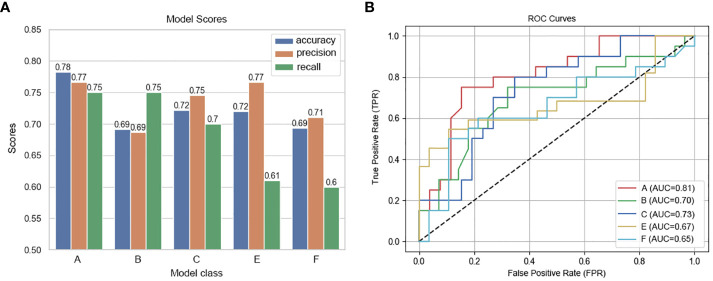
Performance metrics and ROC curves of predictive models across CT imaging phases. **(A)** Model Scores: This bar chart compares the accuracy, precision, and recall of predictive models developed for different phases of contrast-enhanced CT scans. Each model corresponds to a specific phase: Phase A (pretherapeutic plain), Phase B (pretherapeutic venous), Phase C (preoperative arterial), and Phases E and F (preoperative venous), revealing variations in their predictive performance. **(B)** ROC Curves of the Predictive Models Across CT Imaging Phases: This graph displays the ROC curves for each predictive model, with the true positive rate plotted against the false positive rate for various threshold settings.

In this study, we delineated the ROI of the plain phase, arterial phase, and venous phase of contrast-enhanced CT before treatment, as well as the plain phase, arterial phase, and venous phase before surgery, and respectively named these six regions of interest A, B, C, D, E, and F. Due to the absence of complete three-temporal phase data for part of patients in the PACS, and the limited sample size for the preoperative plain phase rendering it unsuitable for feature analysis, preoperative plain phase (D) was excluded. In order to select important features that influence clinical severity, we first investigated the contribution of each of the 107 input variables of each phase on severity via feature importance analysis using XGBoost algorithms. we recursively remove the features with lower importance to obtain smaller feature subsets. We found the optimal feature subset by 5-fold cross-validation. The specific radiomics model parameters used to reduce weights and control overfitting are shown in **Table 2**. Due to the lack of a clear correlation between RFs of the arterial phase before treatment (B) and the outcome pCR, both feature selection and model parameters were unstable and not listed in the table. Finally, we averaged the importance values for the final ranked feature importance value. The features selected based on importance in the radiomics model of the contrast-enhanced CT plain phase before treatment were GLCM_JointEntropy, GLCM_DifferenceAverage, GLDM_DependenceEntropy, Firstorder_Skewness, Firstorder_RootMeanSquared, GLSZM_SizeZoneNonUniformityNormalized, and NGTDM_Complexity. A suite of features including GLDM_DependenceNonUniformity, Firstorder_TotalEnergy, GLCM_MCC, Shape_SurfaceArea, GLSZM_SizeZoneNonUniformity, GLSZM_GrayLevelNonUniformity, and GLSZM_ZonePercentage were also chosen based on importance in the radiomics model of the pretherapeutic contrast-enhanced CT venous phase. In the radiomics model of the arterial phase before surgery, we selected Firstorder_Skewness, GLCM_ClusterShade, GLCM_InverseVariance, Firstorder_Energy, Shape_Maximum2DDiameterColumn, GLCM_Correlation, Shape_LeastAxisLength, Firstorder_RootMeanSquared, and Shape_MinorAxisLength. Similarly, Firstorder_MeanAbsoluteDeviation, Shape_Maximum2DDiameterColumn, Firstorder_RobustMeanAbsoluteDeviation, Shape_MinorAxisLength, GLSZM_ZoneVariance, GLRLM_RunVariance, NGTDM_Busyness, GLCM_Correlation, and GLDM_DependenceNonUniformityNormalized were opted based on feature importance in the radiomics model of the preoperative contrast-enhanced CT venous phase. The performance of radiomics model was gauged through accuracy, precision, recall (**Figure 2A**), and AUC (**Figure 2B**) metrics across different phases. The analysis for phase A revealed optimal predictive power with an AUC of 0.81 and accuracy of 0.78 when seven features were selected. Phase B, with unstable feature selection, achieved an AUC of 0.70 and accuracy of 0.69. Phase C, with seven selected features, reached an AUC of 0.73 and accuracy of 0.72. For phases E and F, the optimal AUC values were 0.67 and 0.65, with accuracies of 0.72 and 0.69 respectively. Ultimately, the radiomics model of the pretherapeutic plain phase (A) was deemed superior.

**Table 2:** Radiomics model parameters of each contrast-enhanced CT phase model.

Phase model	pretherapeutic plain phase	pretherapeutic venous phase	preoperative arterial phase	preoperative venous phase
learning_rate	0.11	0.12	0.09	0.17
min_child_weight	2.65	0.22	2.95	0.54
max_depth	13	14	8	11
max_delta_step	3.46	2.81	2.98	0.02
subsample	0.83	0.79	0.60	0.75
colsample_bytree	0.91	0.84	0.76	0.99
colsample_bylevel	0.83	0.88	0.95	0.90
reg_lambda	0.55	0.33	0.61	0.82
reg_alpha	0.33	0.32	1.00	0.04

Clinicopathological and laboratory data was analyzed based on feature importance in the same way. The combination of clinicopathological characteristics and hematological indicators cannot establish a model-based ranking, as the amount of data is small, and the ranking would easily change with parameters. Eventually, after repeated training, the selected characteristics consist of post-HALP, pathological differentiation, post-LDH, ΔHB, post-APC, ΔPALB, prePLR, and post-ANC.

### 3.3 Joint model

**Figure 3:**
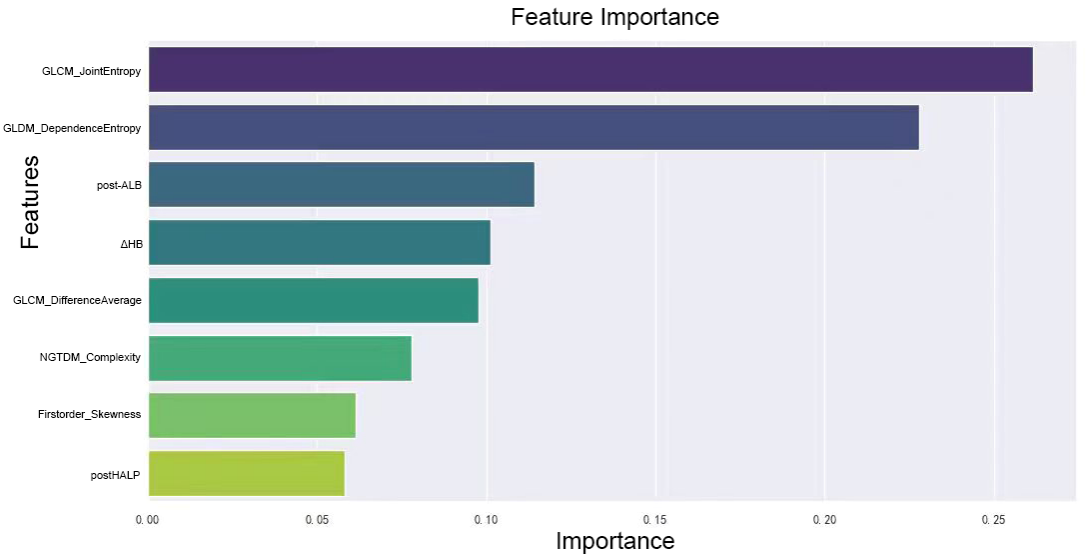
Ranking of feature importance in clinical-radiomic model. This figure displays the relative importance of various radiomic and clinical features as determined by the XGBoost algorithm in our predictive model. Features like GLCM_JointEntropy and GLDM_DependenceEntropy top the chart, emphasizing the significance of tumor textural heterogeneity and internal structure in predicting the response to neoadjuvant therapy. Clinical markers such as post-treatment albumin and changes in hemoglobin levels also feature prominently, highlighting their influence on treatment efficacy, alongside measures of tumor density contrast and complexity. This graph underscores the intricate relationship between radiologic and clinical factors in determining therapeutic outcomes.

**Figure 4:**
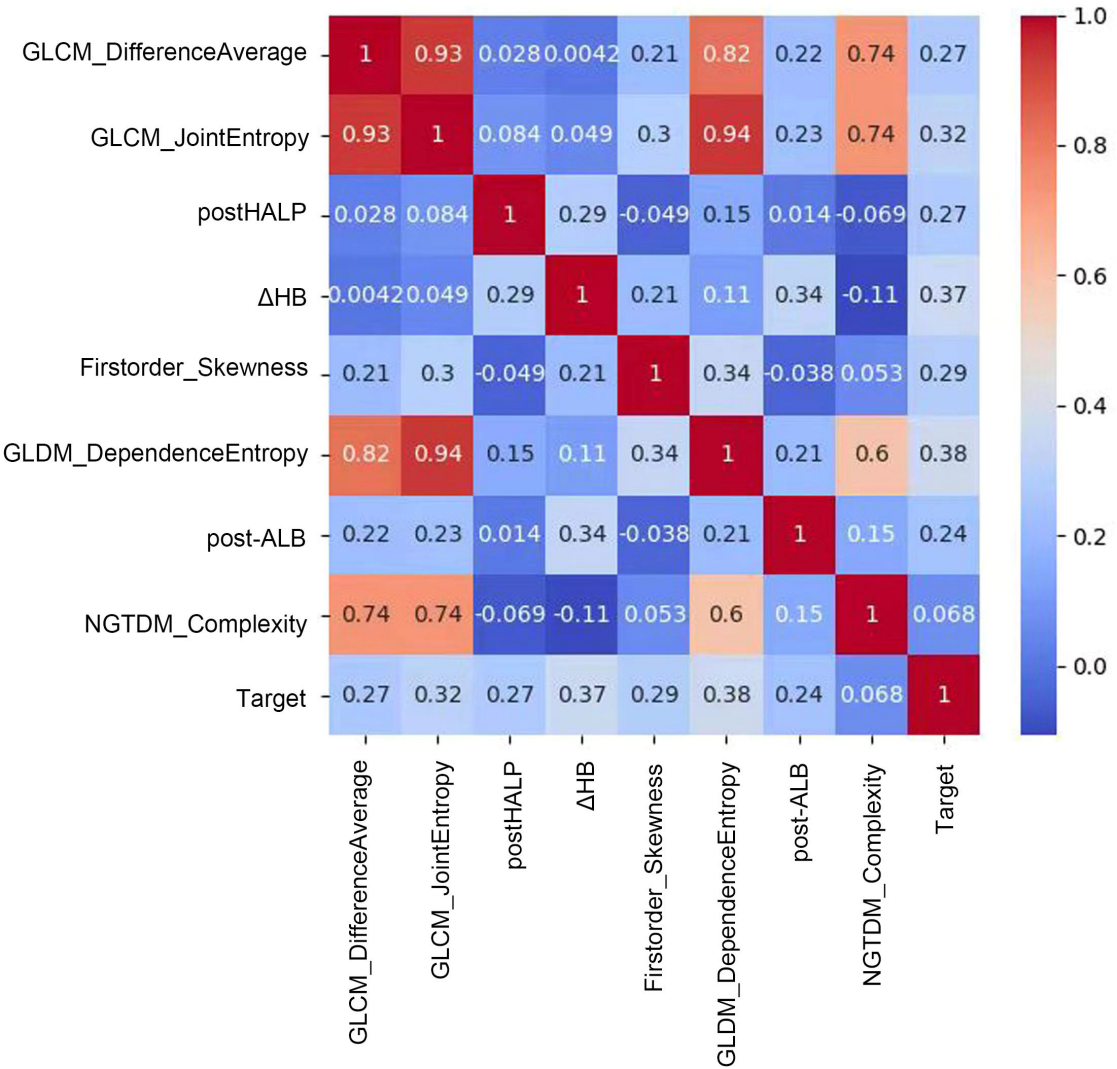
Correlation matrix of radiomic and clinical features in predictive modeling. This figure showcases a correlation matrix that quantifies the relationships between radiomic features and clinical variables in our predictive model. Each cell in the matrix represents the correlation coefficient between feature pairs, indicated by a color gradient from blue (low correlation) to red (high correlation). Notably, a strong positive correlation is observed between GLCM_DifferenceAverage and GLCM_JointEntropy, suggesting a link in tumor texture attributes. Moderate correlations are seen between features like GLDM_DependenceEntropy and the predictive Target, while features such as post-HALP exhibit lower correlation values. This matrix provides insights into which features might combine effectively to enhance the model and which offer unique predictive information, aiding in the development of a nuanced, multidimensional predictive tool for clinical use.

We combined these selected radiomics features of the preoperative contrast-enhanced CT plain phase, clinicopathological characteristics, and hematological indicators for reanalysis. These features included GLCM_JointEntropy, GLCM_DifferenceAverage, GLDM_DependenceEntropy, Firstorder_Skewness, Firstorder_RootMeanSquared, GLSZM_SizeZoneNonUniformityNormalized, NGTDM_Complexity, post-HALP, pathological differentiation, post-LDH, ΔHB, post-APC, ΔPALB, prePLR, and post-ANC. Because the combination of the two may cause new feature redundancy, the XGBoost algorithm was used to perform feature importance analysis to remove features with low importance. Five-fold cross-validation was employed to score different feature subsets and select the best-scoring set of features. The model identified crucial predictors. These were postHALP, ΔHB, post-ALB, firstorder_Skewness, GLCM_DifferenceAverage, GLCM_JointEntropy, GLDM_DependenceEntropy, and NGTDM_Complexity. As shown in **Figure 3**, GLCM_ JointEntropy contributed the most in the joint model to predict the outcome, followed by GLDM_DependenceEntropy, and postHALP had the smallest contribution. Concurrently, the overarching trend of the joint model was observed to possess a positive correlation, as highlighted in **Figure 4**.

**Figure 5:**
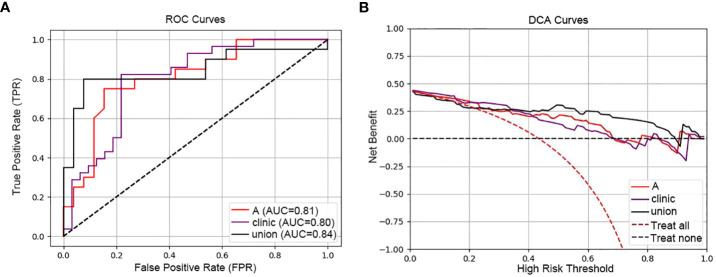
ROC and DCA curves comparing predictive models. [**(A)** ROC curves of the three models (radiomic model, clinical model and clinical-radiomic model)]: This panel presents the Receiver Operating Characteristic (ROC) curves for three models: the radiomic model **(A)** with an AUC of 0.81, the clinical model (Clinic) with an AUC of 0.80, and the combined clinical-radiomic model (Union) with an AUC of 0.84, demonstrating that combining radiomic and clinical data provides the most accurate prediction of treatment response. **(B)** DCA curves of the three models (radiomic model, clinical model and clinical-radiomic model)): The Decision Curve Analysis (DCA) for the same models illustrates the net benefits across a spectrum of risk thresholds. The combined model (Union) shows the highest net benefit, suggesting its greater clinical value in making informed treatment decisions for esophageal cancer.

**Figure 5A** contrasts the predictive performances of clinicopathological and hematological analyses, radiomics, and clinical-radiomic model. With an AUC of 0.84 for the joint model, it outperformed the radiomics (AUC 0.81) and clinical model (AUC 0.80). The joint model also exhibited superior clinical applicability compared to individual analyses, as depicted in **Figure 5B**.

## 5 Discussion

In patients with resectable disease, neoadjuvant chemoradiotherapy (nCRT) combined with esophagectomy remains the primary treatment. However, the pCR rate for nCRT, which ranges between 29.2% to 43.2%, is not entirely satisfactory. ICIs have dramatically shifted the treatment paradigm for numerous advanced cancers, EC included. Recent evidence has suggested that neoadjuvant immunotherapy can potentially boost survival rates in patients with resectable cancers. Early clinical trials assessed neoadjuvant immunotherapy in tandem with chemoradiotherapy, with most of these studies originating from China and focusing on ESCC. Yet, neoadjuvant immunotherapy in EC is still nascent, and many questions linger. One challenge is in assessing the response. Pathological response frequently acts as the surrogate endpoint for both relapse-free survival and overall survival in neoadjuvant cancer therapy (39). Currently, pCR and MPR are the preferred metrics for gauging response to neoadjuvant immunotherapy.

In our study, we enrolled 60 ESCC patients who underwent ICRT. We delved into the correlation between hematologic indicators, clinicopathological traits, and radiomics features extracted from the primary tumor’s region across various CT phases, and prognosis in ESCC patients undergoing nICRT. Our findings indicated that postHALP, ΔHB, post-ALB, firstorder_Skewness, GLCM_DifferenceAverage, GLCM_JointEntropy, GLDM_DependenceEntropy, and NGTDM_Complexity are potential biomarkers to forecast pCR. The general trend from the joint model appears to be positively inclined. Previous research has corroborated that a rise in petherapeutic inflammatory biomarkers significantly aligns with the prognosis for esophageal cancer patients who have undergone either curative esophagectomy or definitive chemoradiotherapy (40–45). Yet, for those treated with nICRT, the relevant studies are scant. One notable study aimed to create and validate a predictive model, the integrative inflammatory and nutritional score (IINS), for locally advanced esophageal squamous cell carcinoma patients receiving neoadjuvant immunotherapy combined with chemotherapy (nICT), to project the pCR. This was devoid of radiomics correlation (46). Furthermore, most research predicting neoadjuvant efficacy for esophageal cancer primarily harnesses enhanced CT or 18F-FDG-PET-CT to predict pCR following neoadjuvant therapy (27, 47, 48). Given that therapeutic effect is a dynamic process, the data involving RFs changes during treatment would be more informative. Moreover, there are differences in RFs due to the uptake of contrast agents during the different phases of contrast-enhanced CT. Consequently, we introduced longitudinal images to assess the connection between RFs and outcomes, including pre- and post- neoadjuvant treatment, and to enhance clinical usefulness further. Our joint model not only encompasses clinical features, laboratory metrics, and contrast-enhanced CT evaluations but also extends CT analysis based on phase. Both hematologic biomarkers, sourced from peripheral blood, and CT emerge as straightforward and accessible tools to anticipate the prognosis for ESCC patients. We posit that the clinical-radiomic assessment in our study offers promising diagnostic markers to predict postoperative pathological outcomes in EC patients treated with nICRT regarding pCR achievement.

We endeavored to predict pCR quantitatively using radiomics features from plain, arterial, and venous phase CT, targeting the identification of the phase with the most significant applications. To mitigate complexity and overfitting, we deployed the XGBoost algorithm for feature selection. Prior CT radiomics investigations were singularly based on either one temporal or one level of RFs, rendering the attainment of the optimal model for specific clinical concerns elusive (49). Our feature extraction rooted in a three-dimensional ROI is arguably more adept at delineating tumor spatial heterogeneity compared to a single layer. In addition, we leveraged richer RFs from multi-temporal CT, promoting a comprehensive analysis of the association between images and disease mechanisms. Interestingly, the AUC value under the ROC of the plain phase displayed an uptick. We conjecture this is attributed to tumor heterogeneity. Tumor heterogeneity is a hallmark of malignancies, directly influencing their growth rate, invasive capacity, drug sensitivity, and prognosis (50). Evidence suggests that tumor heterogeneity’s nuances are challenging to encapsulate through a singular method. However, it can be quantitatively explored through RFs (25). The plain phase meticulously chronicles the intensity and spread of all RFs within the tumor. Post-enhancement, with the influx of positive contrast agents into the tumor’s blood vessels, some may be absorbed by tumor cells. While this may shed light on specific other tumor biological behaviors, it also alters the tumor’s inherent texture feature distribution, particularly grayscale intensity, hence, modifying its predictive capacity for nICRT’s efficacy in esophageal cancer. This could potentially clarify the reduced predictive performance seen in arterial and venous phases. Numerous machine algorithms are available for radiomics model, including mainstream ones like random forest, logistic regression, support vector machine, and decision tree. The choice of the appropriate algorithm is pivotal for constructing the model. Our study employed a decision tree algorithm to predict the efficacy of neoadjuvant immunotherapy combined with chemoradiotherapy for esophageal squamous cell carcinoma.

Nevertheless, our study has its limitations. Firstly, the sample size might be considered modest, which could introduce bias. Incorporating a larger sample size would undoubtedly enhance and authenticate its utility as a valuable prediction tool to aid treatment decisions. Secondly, our conclusions stem from a retrospective design. The joint model’s performance in prospective studies awaits exploration. Lastly, owing to CT’s constrained spatial resolution, discerning the demarcation between the lesion and regular esophageal tissue during ROI segmentation might be susceptible to bias. Further advancements in medical imaging techniques and precision are imperative. Thus, in subsequent research, our ambition is to acquire multi-center data, bolster the sample size, and undertake radiomics feature extraction more scientifically and effectively, enhancing the predictive capability and clinical applicability of the predictive model. Our study, at this juncture, provides insights for devising novel strategies to assess the efficacy in locally advanced resectable esophageal cancer patients post-nICRT followed by surgical intervention, undoubtedly supplementing the existing predictive tools.

## 6 Conclusion

In summation, our clinical-radiomic model, anchored on postHALP, ΔHB, post-ALB, firstorder_Skewness, GLCM_DifferenceAverage, GLCM_JointEntropy, GLDM_DependenceEntropy, and NGTDM_Complexity, seeks to predict pathologic complete response. Our aspiration is to present an alternative tool to identify prospective best responders to nICRT prior to the commencement of treatment for ESCC patients, thereby aiding clinical decision-making.

## Data availability statement

The original contributions presented in the study are included in the article/**Supplementary Material**. Further inquiries can be directed to the corresponding author.

## Ethics statement

The studies involving humans were approved by Ethics Committee of Shandong Cancer Hospital. The studies were conducted in accordance with the local legislation and institutional requirements. The ethics committee/institutional review board waived the requirement of written informed consent for participation from the participants or the participants’ legal guardians/next of kin because this is a retrospective study that does not involve the personal privacy of patients and poses no risk to the subjects. The experimental results are only for scientific research purposes. Based on the above situation, we applied to the committee to waive the informed consent of the subjects.

## Author contributions

XW: Data curation, Formal analysis, Investigation, Validation, Visualization, Writing – original draft, Writing – review & editing. GG: Methodology, Writing – review & editing. QS: Resources, Writing – review & editing. XM: Conceptualization, Funding acquisition, Project administration, Writing – review & editing.
