# Supplementary material for: Prediction of pCR based on clinical-radiomic model in patients with locally advanced ESCC treated with neoadjuvant immunotherapy plus chemoradiotherapy
Source: Front Oncol. 2024 Mar 20;14:1350914. doi: 10.3389/fonc.2024.1350914 (PMC10989074; doi:10.3389/fonc.2024.1350914)
Supplement: Supplementary file 1 [file Table_1.docx]

**Supplementary Table S1.**

Hematological parameters of the patients. Values are presented as median (IQR). Abbreviations: pCR, pathologic complete response; LDH, lactate dehydrogenase; ALB, albumin; WBC, white blood cell count; ANC, absolute neutrophil count; ALC, absolute lymphocyte count; AMC, absolute monocyte count; APC, absolute platelet count; HB, hemoglobin; PAB, prealbumin; NLR, neutrophil to lymphocyte ratio; PLR, platelet to lymphocyte ratio; MLR, monocyte to lymphocyte ratio. P value is calculated from two-sample t-test/Mann-Whitney U test for continues variables, which represents the univariate association test of subgroups. P Between responders and non-responders in the total set.

| Characteristic | pCR  (N=28) | Non-pCR  (N=32) | p-value |
| --- | --- | --- | --- |
| Pre-LDH (U/L) | 190.00  (180.24-202.26) | 192.50  (178.82-202.93) | 0.963 |
| Pre-ALB (g/L) | 44.10  (42.63-48.29) | 45.70  (43.23-46.13) | 0.603 |
| Pre-WBC (×10^9^/L) | 6.66  (6.20-7.64) | 6.35  (6.05-7.44) | 0.720 |
| Pre-ANC (×10^9^/L) | 4.06  (3.79-5.23) | 4.01  (3.76-5.05) | 0.820 |
| Pre-ALC (×10^9^/L) | 1.70  (1.54-1.91) | 1.60  (1.50-1.79) | 0.508 |
| Pre-AMC (×10^9^/L) | 0.46  (0.42-0.53) | 0.48  (0.43-0.57) | 0.564 |
| Pre-APC (×10^9^/L) | 246.00  (232.76-276.88) | 274.50  (248.72-298.22) | 0.261 |
| Pre-HB (g/L) | 145.50  (138.60-149.33) | 149.50  (142.49-155.19) | 0.242 |
| Pre-PALB (g/L) | 0.24  (0.22-0.27) | 0.28  (0.25-0.30) | 0.118 |
| Pre-NLR | 2.41  (2.23-3.49) | 2.59  (2.333-3.30) | 0.912 |
| Pre-PLR | 152.47  (138.18-174.48) | 177.53  (155.02-195.58) | 0.164 |
| Pre-MLR | 0.26  (0.25-0.34) | 0.28  (0.27-0.37) | 0.487 |
| Pre-SII | 574.9  (564.87-874.08) | 676.78  (627.82) | 0.552 |
| Pre-SIRI | 1.07  (0.97-1.91) | 1.11  (1.05-2.06) | 0.739 |
| Pre-PNI | 51.97  (50.98-57.18) | 52.70  (51.11-54.71) | 0.493 |
| Pre-HALP | 41.41  (38.78-54.67) | 39.88  (36.59-50.66) | 0.552 |
| Post-LDH (U/L) | 207.00  (192.56-236.30) | 179.00  (178.68-214.51) | 0.198 |
| Post-ALB (g/L) | 41.25  (38.64-43.10) | 39.90  (38.49-41.20) | 0.412 |
| Post-WBC (×10^9^/L) | 5.67  (5.34-8.29) | 4.89  (4.67-6.38) | 0.115 |
| Post-ANC (×10^9^/L) | 3.02  (2.88-5.66) | 3.19  (2.95-4.71) | 0.578 |
| Post-ALC (×10^9^/L) | 1.06  (0.68-3.00) | 0.90  (0.85-1.10) | 0.107 |
| Post-AMC (×10^9^/L) | 0.49  (0.47-0.70) | 0.49  (0.46-0.56) | 0.293 |
| Post-APC (×10^9^/L) | 193.50  (181.29-211.43) | 219.00  (205.47-254.53) | 0.025 |
| Post-HB (g/L) | 127.00  (123.82-137.32) | 121.50  (119.47-127.78) | 0.070 |
| Post-PALB (g/L) | 0.26  (0.23-0.28) | 0.26  (0.21-0.26) | 0.329 |
| Post-NLR | 3.13  (2.51-12.22) | 2.69  (2.71-8.53) | 0.518 |
| Post-PLR | 199.98  (165.99-288.98) | 243.38  (215.59-344.87) | 0.231 |
| Post-MLR | 0.43  (0.41-1.01) | 0.55  (0.49-0.71) | 0.470 |
| Post-SII | 572.69  (501.45-2326.66) | 712.28  (641.52-2052.60) | 0.905 |
| Post-SIRI | 1.21  (0.68-9.98) | 1.40  (1.58-4.14) | 0.270 |
| Post-PNI | 48.10  (44.05-56.11) | 43.92  (43.07-46.36) | 0.067 |
| Post-HALP | 29.85  (20.40-76.37) | 21.35  (18.60-28.43) | 0.061 |
| Delta LDH (U/L) | 4.50  (-0.76- 47.12) | 1.50  (-10.89- 22.33) | 0.216 |
| Delta ALB (g/L) | -3.90  (-8.20- -0.97) | -5.25  (-6.78- -2.87) | 0.901 |
| Delta WBC (×10^9^/L) | -1.42  (-1.84-1.63) | -1.86  (-2.24- -0.20) | 0.247 |
| Delta ANC (×10^9^/L) | -0.82  (-1.76-1.28) | -1.09  (-1.53-0.38) | 0.699 |
| Delta ALC (×10^9^/L) | -0.63  (-1.03-1.27) | -0.62  (-0.78- -0.56) | 0.139 |
| Delta AMC (×10^9^/L) | 0.01  (-0.02-0.21) | 0.02  (-0.05-0.08) | 0.203 |
| Delta APC (×10^9^/L) | -51.50  (-81.26- -35.67) | -46.50  (-64.24- -22.70) | 0.323 |
|  |  |  |  |
| Delta HB (g/L) | -11.00  (-19.16- -7.63) | -23.50  (-31.76- -18.68) | 0.008 |
| Delta PALB (g/L) | 0.00  (-0.02-0.04) | -0.03  (-0.06- -0.01) | 0.015 |
